# Antibiotics in Raw Meat Samples: Estimation of Dietary Exposure and Risk Assessment

**DOI:** 10.3390/toxics10080456

**Published:** 2022-08-06

**Authors:** Athina Stavroulaki, Manolis N. Tzatzarakis, Vasiliki Karzi, Ioanna Katsikantami, Elisavet Renieri, Elena Vakonaki, Maria Avgenaki, Athanasios Alegakis, Miriana Stan, Matthaios Kavvalakis, Apostolos K. Rizos, Aristidis Tsatsakis

**Affiliations:** 1 Laboratory of Toxicology, Medicine School, University of Crete, 70013 Heraklion, Greece; 2 Department of Chemistry, University of Crete and Foundation for Research and Technology—Hellas (FORTH-IESL), 70013 Heraklion, Greece; 3 Department of Toxicology, Faculty of Pharmacy, Carol Davila University of Medicine and Pharmacy, 200349 Bucharest, Romania

**Keywords:** tetracyclines, sulfonamides, quinolones, streptomycines, meat, antibiotics, risk assessment

## Abstract

The extensive use of antibiotics in livestock farming poses increased concerns for human health as residues of these substances are present in edible tissues. The aim of this study was the determination of the levels of four groups of antibiotics (sulfonamides—SAs, tetracyclines—TCs, streptomycines—STr and quinolones—QNLs) in meat samples (muscles, livers and kidneys from beef, chicken and pork) and the estimation of the dietary exposure to antibiotics from meat consumption and the potential hazard for human health. Fifty-four samples of raw meat were randomly collected in 2018 from the Cretan market, Greece and analyzed both with an enzyme-linked immunosorbent assay (ELISA) and liquid chromatography–mass spectrometry (LC–MS). According to the results derived from the ELISA method, only 2% of the meat samples were free from antibiotics, 2% were detected with 4 antibiotics and the great majority of the samples (87%) were detected with 2 to 3 antibiotics. SAs presented the highest detection frequencies for all samples whereas TCs were not detected in any bovine sample. The highest median concentration was detected for STr in bovine muscles (182.10 μg/kg) followed by QNLs (93.36 μg/kg) in pork kidneys whereas the chicken samples had higher burdens of QNLs compared to the other meat samples. LC–MS analysis showed that oxytetracycline (OTC) was the most common antibiotic in all samples. The highest median concentration of all antibiotics was detected for doxycycline (DOX) (181.73 μg/kg in pork kidney) followed by OTC in bovine liver (74.46 μg/kg). Risk characterization was applied for each of the two methods; The hazard quotients (HQ) did not exceed 0.059 for the ELISA method and 0.113 for the LC–MS method for any group of antibiotics, whereas the total hazard indexes (HI) were 0.078 and 0.021, respectively. The results showed the presence of different groups of antibiotics in meat from the Cretan market and that the health risk to antibiotics is low. A risk assessment analysis conducted for meat consumption and corrected for the aggregated exposure revealed no risk for the consumers.

## 1. Introduction

The aim of antibiotics is to destroy bacteria and they are used in livestock and poultry production for therapeutic purposes to prevent, control and treat infectious diseases in animals, although some producers use antibiotics to improve meat production by increasing the rate of animal growth [1]. Antibiotics as growth promoters are no longer used in European Union countries as there has been a legal ban from January 2006 [2]. The widespread and prolonged use of antibiotics has contributed negatively to their effectiveness and thus the doses have been increased, alternative more powerful antibiotics have to be used and the times of administration have to be extended [3]. In cases where antibiotics are misused and legal withdrawal periods (the time span from drug administration to animal slaughter and use of meat for human consumption) are not respected, the residues in edible tissues pose an increased risk for consumers [4].

The parent substance of the antibiotics poses the highest toxicity; however, in the human it is metabolized and converted into an inactive and more easily excreted form [5,6]. Allergic reactions and other toxic effects have been observed and the risk is greater for hypersensitive individuals. The most common health effects of quinolones (QNLs) include effects on the central nervous system (CNS), such as anxiety, worry, nervousness and dizziness [7]. In addition to seizures, other serious CNS reactions include delirium, delusions, psychosis, mania, encephalopathy and dysarthria [8]. Recently, pharmacovigilance studies found a possible association between QNLs and peripheral nervous system toxicity [9], including Guillain–Barré syndrome (GBS), a potentially severe form of acute peripheral polyneuropathy [10]. In 2012, a study by a Canadian research team showed an increased risk of retinal detachment associated with the oral administration of QNLs [11]. Gastrointestinal symptoms such as indigestion, nausea, vomiting and diarrhea are common side effects associated with QNL consumption [12].

Allergic reactions associated with sulfonamides (SAs) include the full range of Gell–Coombs hypersensitivity reactions. In addition, there are reactions associated with immunoglobulin E (IgE), such as urticaria, angioedema and anaphylaxis [13]. SAs have been correlated with hepatotoxicity and systemic hypersensitivity reactions [14,15].

Tetracyclines (TCs) can modify the normal intestinal flora, allowing the overproduction of Pseudomonas and Clostridium [16], and cause nausea, diarrhea and even mortality. They are also found in the structure of newly formed teeth, if consumed during certain periods of pregnancy, such as the embryonic period (from the third through the eighth week after conception) [17]. Hepatotoxicity occurs in patients with hepatic impairment or after intravenous administration of TCs and nephrotoxicity when administered concomitantly with diuretics [18].

Streptomycines (STr) belong to the aminoglycosides (AGs) category of antibiotics. Patients receiving AGs may have reversible nephrotoxicity [19] because AGs can enter the proximal tubule through megaline, a multiligand binding receptor. AG excretion from this intracellular compartment occurs very slowly and can take several days [20]. Side effects include cochlear damage of the auditory nerve [21], optic nerve dysfunction [22], peripheral neuropathy [23], arachnoiditis [24] and encephalopathy [25].

Μeat and dairy products constitute an important part of the diet. In 2013, global poultry meat production exceeded 109 million tons and global egg production was estimated at over 73 million tons. In 2014, global production of beef and pork was estimated at about 170 million tons [26]. A major review by the Food and Agriculture Organization (FAO) of the United Nations, which makes extensive use of expert judgement, reported an increase of 76% in the total quantity of meat consumed by the mid-century. This includes a doubling in the consumption of poultry, a 69% increase in beef and a 42% increase in pork [27]. In Europe, cheese and pig meat are the preferred animal-based protein sources, followed by poultry, milk and bovine meat. The EU citizen consumed an average of 2.2 kg less bovine meat in 2013 than in 2000 (decreased by 13%), but 3.0 kg more poultry (increased by 15%). Pork consumption remained nearly fixed throughout this period. According to FAOSTAT (Food and Agriculture Organization of the United Nations) [28], in Greece the mean consumption of bovine meat in 2019 was 14.1 kg/capita/year, for pork 28.9 kg/capita/year and for poultry 25.6 kg/capita/year.

As noted by Arsène et al., antibiotic residues in food, such as meat, are likely to induce antibiotic resistance in bacteria and cause allergies and other more severe effects in humans [29]. This fact, combined with the high positivity in food samples, leads to the assumption that increased meat consumption may be associated with a risk of antibiotic contamination. In addition, as the European Medicines Agency (EMA) describes, when the withdrawal period (“The time that must elapse between the last administration of a veterinary medicine and the slaughter or production of food from that animal”) is not respected then the antibiotic residues in meat can exceed the maximum residue levels (MRLs) [30].

This study aims at screening the antibiotic residues in bovine, pork and chicken samples (muscle, liver and kidney) from the local Cretan market, assessing the exposure of the Cretan population to certain compounds due to meat consumption and ultimately estimating the risk for human health resulting from the dietary intake of multiple antibiotics through meat consumption, corrected for the aggregated dietary exposure.

## 2. Materials and Methods

### 2.1. Reagents

Methanol (99.9%), formic acid (≥95%) and acetonitrile (≥99.9%) were purchased from Honeywell. Ethyl acetate (99.8%), NaCl (99.9%), n-hexane (99%) and phosphate buffer saline (PBS) tablets were from Sigma Aldrich (Saint Louis, MO, USA). Ultrapure water (Direct-Q 3UV), Na_2_HPO_4_ × 2H_2_O (99.5%) and NaOH (99%) were purchased from Merck (Darmstadt, Germany). ELISA kits (R3505 RIDASCREEN^®^ Tetracyclin, R3004 RIDASCREEN^®^ Sulfonamide, R3104 RIDASCREEN^®^ Streptomycin, R3113 RIDASCREEN^®^ Quinolones) were purchased from R-Biopharm (Darmstadt, Germany).

### 2.2. Sampling

A total of 54 samples of raw meat were randomly collected on November 2018 from butcheries in Crete, Greece. The samples were collected from the area of Crete but the animals originated from all over the country. Data concerning the age of the animals and the country of origin were collected. The collected samples were 16 (29.6%) bovine samples, 20 (37.0%) chicken and 18 (33.3%) pork. The collected samples consisted of 29 muscles (53.7%), 17 livers (31.5%) and 8 kidneys (14.8%). Out of the 29 samples there were 10 beef muscles, 6 beef livers, 10 pork muscles, 2 pork livers, 6 pork kidneys, 9 chicken muscles, 9 chicken livers and 2 chicken kidneys. Beef kidneys were not found in any Cretan butcher shop. The majority of the samples (81.5 %) came from animals of Greek origin. The average age of cattle was 15.5 ± 3.3, for pork 4.9 ± 2.0 and for chicken 2.3 ± 0.8 months. All samples were weighted and packed in properly labeled conical centrifuge tubes, sealed and kept at −20 °C, until the analysis.

### 2.3. Sample Preparation

Total SAs, TCs, STr and QNLs residues were detected using an ELISA test kit. The samples were cut into small pieces and then homogenized with a homogenizer of Janke & Kunkel, Ultraturrax T25 (Staufen, Germany). Then, they were placed in 50 mL Falcon tubes and stored in the freezer (−20 °C) until use. The sample preparation and analysis protocols were instructed from the manufacturer. Briefly, for SAs determination, the homogenized samples were weighed (1 g pork/bovine, 2 g chicken) and vortexed with organic solvent (2 mL methanol for pork/bovine, 6 mL acetonitrile/water 84:16 *v*/*v* for chicken). The mixture was centrifuged at 4000 rpm for 10 min and an aliquot of 1.5 mL of supernatant was evaporated to dryness. The dry residue was reconstituted in 0.5 mL buffer (provided by the kit) and 1 mL n-hexane was added. An aliquot of 50 μL of the lower phase was used for analysis. For chicken samples, 4 mL of the supernatant were transferred into a new centrifuge vial, 2 mL 2 M NaCl and 7 mL ethyl acetate were added and the mixture was shaken for 10 min. The mixture was centrifuged for 10 min at 3000 rpm (15 °C). The whole supernatant was evaporated to dryness and reconstituted in 1 mL sample buffer and 1 mL n-hexane. An aliquot of 50 μL of the lower phase was used for analysis.

For STr, 5 g of homogenized sample were mixed with 20 mL of wash buffer, vortexed for 10 s and shaken for 30 min. The mixture was centrifuged (10 min, 4000 rpm, 25 °C), the supernatant was diluted with wash buffer (1:10) and 50 μL were used for analysis.

For TCs, 1 g of homogenized sample and 9 mL 20 mM PBS buffer pH 7.4 were transferred into a centrifuge vial and shaken 10 min for extraction. Then, the mixture was centrifuged (10 min, 4000 rpm, 25 °C) and 1 mL of supernatant was transferred and mixed with 2 mL of n-hexane. An aliquot of 50 µL of the lower aqueous phase was used per well in the assay.

For QNLs, 1 g of homogenized sample and 4 mL methanol/water (70/30, *v*/*v*) were mixed vigorously for 10 min and centrifuged (10 min, 4000 rpm, 25 °C). The supernatant was diluted with washing buffer (1:2) and 50 µL were used for analysis.

After samples/standards were loaded, 50 μL of antibody solution were added in each well and plates were incubated for 1 h at room temperature. The wells were washed with 250 μL buffer three times, 100 μL of substrate/chromogen was added and incubated for 15 min at room temperature in the dark. Finally, 100 µL of the stop solution were added to each well and the absorbance was measured at 450 nm.

The LC–MS-based methodology for the detection of antibiotics residues was carried out according to a previously published method [31]. Briefly, 500 µL of EDTA 150 mM were added in 5 g of homogenized meat and vortexed for 10 minutes. Extraction was carried out with 5 mL acidified acetonitrile (0.1% formic acid) for 10 minutes and then the mixtures were placed in the freezer (−20 °C) for 30 minutes. Then extracts were centrifuged (10 min, 4000 rpm), the supernatant was collected and the extraction was repeated. The combined supernatants were evaporated to dryness and the dry residue was reconstituted in 500 µL of the mobile phase.

### 2.4. Instrumental Analysis

A Shimadzu LC-MS-2010EV (Kyoto, Japan) was used for the detection and quantification of the analytes after the separation of the analytes on a Supelco Discovery C18 column (25 cm × 4.6 mm, 5 μm) (Sigma-Aldrich, Saint Louis, MO, USA). The oven was set at 30 °C and the flow rate was 0.6 mL/min. The mobile phase was water with 0.1% formic acid (Solvent A) and acetonitrile with 0.1% formic acid (Solvent B). The mass spectrometer was coupled with an ESI (electrospray ionization) ion source and the detection was achieved in selected ion monitoring (SIM) in positive mode. The retention times and m/z ions were for MBX: 8.66 min and m/z 362.1, for OTC: 8.90 min and m/z 461.15, for ENR: 9.21 min and m/z 360.1, for DOX: 10.48 min and m/z 445.05, for SDZ: 8.01 min and m/z 251.0/272.9 and for SMX: 11.11 min and m/z 254.0/275.9, respectively

### 2.5. Exposure Assessment

Exposure of the general population was assessed for each one of the four antibiotic groups (SAs, TCs, QNLs and STr). The daily dietary intake of antibiotics derives from the antibiotic concentration in food consumed and the daily food consumption.

Consumption data for the Greek population for all food items were retrieved from the FAOSTAT database [28] and 2019 data are represented (Table 1). The estimated daily intake of antibiotics from meat, and specifically bovine meat, pig meat and poultry meat (EDImeat) (μg/kg body weight/day), was calculated using the following equation:

EDImeat = Cantibiotic × Wfood/BW(1)

where cantibiotic is the concentration of antibiotics in meat tissue determined in this study (bovine meat, pig meat and poultry meat), expressed as the median concentration (μg/kg meat, on fresh weight basis), Wmeat (g meat/capita) represents the daily average consumption of meat (bovine meat, pig meat and poultry meat) per person and BW is the mean body weight for an adult consumer (70 kg).

### 2.6. Risk Characterization

Risk characterization was conducted following the approach of the source-related hazard quotient (HQ) and hazard index (HI) initially proposed in Goumenou and Tsatsakis [32], and application of the methodology is presented in details in relevant case studies [33,34,35,36,37]. Using this approach, the source-related hazard quotient (HQ) is assessed, after accounting for the correction factor for meat (CFm). The CFm expresses the contribution of meat to the total antibiotic dietary daily intake and it is equal with the ratio of the maximum permitted daily intake through meat consumption MPDIm (meat consumption × maximum residue level (MRL) in meat) to the maximum permitted daily intake through the whole diet, MPDIA (SUM of MPDIi = SUM (food_i_ consumption × MRL in the food_i_), where food_i_ represents each food item with considerable contribution in the overall exposure).

MPDIA = ΣMPDIi(2)

CFm = MPDIm/MPDIA (3)

More specifically, CFm = (consumption data for the meat × MRL for meat)/SUM (consumption data for relevant food_i_ × MRL in relevant food_i_).

The corrected EDImeat is calculated with the formula:

cEDImeat = EDImeat/CFm(4)

ADI and MRL values in relevant food items were extracted from official databases, such as the European Commission [38] and FAO/WHO [39]. According to FAO/WHO, the ADI for SAs and STr is 50 μg/kg bw/day whereas the corresponding value for tetracyclines is 30 μg/kg bw/day. The ADI for quinolones is referred to as 6.2 μg/kg bw/day and specifically for enrofloxacin, selected as the most conservative value [40]. MRLs for TCs, SAs and QNLs were set to be 100 μg/kg, whereas for STr the MRL is 600 μg/kg. The food groups contributing the most to the dietary antibiotic intake we considered from the FAOSTAT database [28] were: honey, bovine meat, mutton and goat meat, pig meat, poultry meat, meat, other, offals, edible butter, ghee, cream, eggs, milk—excluding butter, freshwater fish, demersal fish, pelagic fish, marine fish, other.

Finally, the source-related hazard quotients (HQs) for each antibiotic group (SAs, TCs, QNLs and STr) were calculated with the following formula

HQ = cEDImeat/ADI(5)

and the HI was calculated as the sum of all HQs.

For considering no risk it should be: CFmi > Hqi, where i is the respective antibiotic group/antibiotic.

## 3. Results

### 3.1. Method Performance

For LC–MS analysis, standard solutions of SMX, SDZ, OTC, DOX, MBX and ENR were prepared at concentrations of 0, 50, 100, 250 and 500 ng/mL. Samples of blank raw meat were used for the preparation of spiked samples at concentrations of 0, 10, 25, 50 and 100 μg/kg. The calibration curves were created by the spiked samples and the coefficient of determination (r^2^) showed good method linearity for all compounds. The mean accuracy ranged from 92.2% (SMX) to 108.9% (OTC). Limits of detection (LODs) were calculated from the signal-to-noise ratio (S/N) which was S/N > 3 and the achieved values ranged from 0.04 μg/kg (ENR) to 2.54 μg/kg (SDZ) depending on the tissue. Likewise, limits of quantification (LOQs) were calculated as S/N > 10 and the values ranged from 0.13 μg/kg (ENR) to 8.38 μg/kg (SDZ) (Table 2).

### 3.2. Antibiotic Concentrations Determined with LC–MS

**Figure 1:**
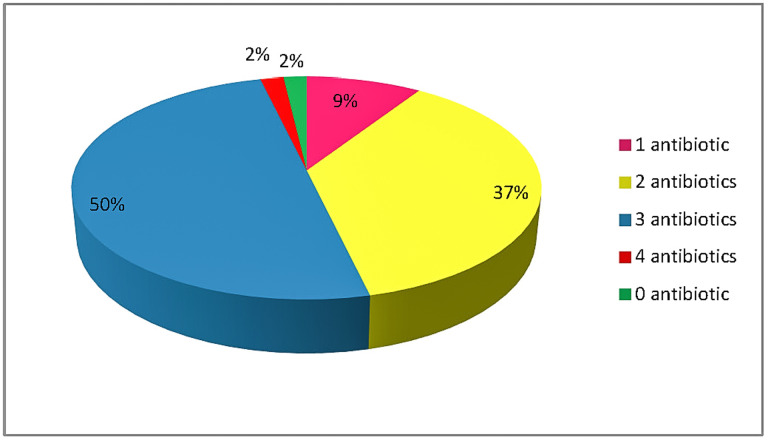
Percentage detection of the investigated antibiotics in all meat samples.

The concentrations of antibiotics that were detected in meat samples by LC–MS are presented in Table 3. The highest median concentrations were detected for DOX at pork kidney (181.73 μg/kg), and OTC at bovine liver (74.46 μg/kg) and chicken liver (64.74 μg/kg). SMX, DOX and MBX were not detected in any bovine liver sample although they were detected in bovine muscle samples. ENR was the one and only antibiotic that was detected in kidneys from chicken at a median concentration of 2.10 μg/kg and it was positive in 100% of the samples. The use of ENR in poultry has been banned by the FDA since 2005 [31], the EU MRL is 100 μg/kg and the detected levels in the present study are lower. According to the results obtained using the ELISA method, only 2% of the meat samples were free from antibiotics, 2% were detected with 4 antibiotics and the great majority of the samples (87%) were detected with 2 to 3 antibiotics (Figure 1).

### 3.3. Antibiotic Concentrations Determined with ELISA

**Figure 2:**
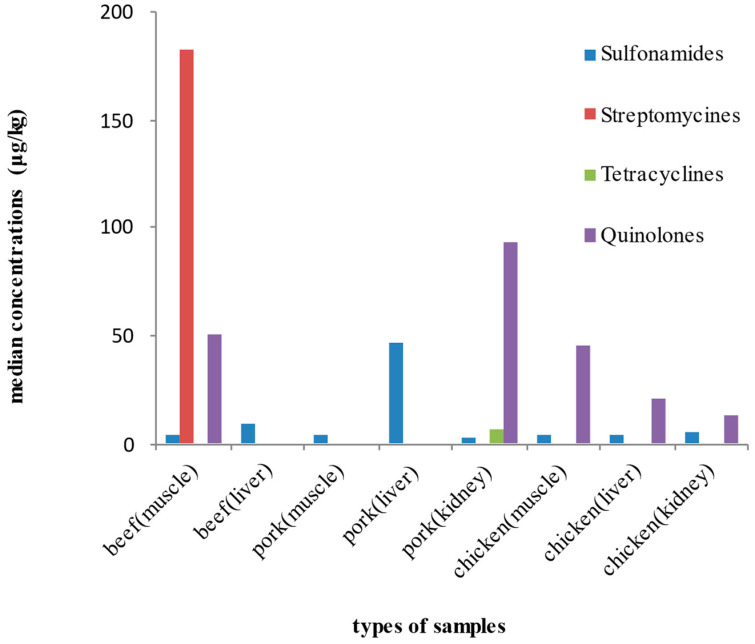
Median concentrations of the investigated antibiotics (μg/kg) determined by the ELISA protocol in all meat samples.

The concentrations of antibiotics that were detected in all meat samples with the ELISA protocol are presented in Table 4 and Figure 2. SAs were the most frequently detected antibiotics in all meat samples, as percentage detection frequency ranged from 83% to 100%. The highest median concentrations were detected in bovine muscles for STr and QNLs at 182.10 μg/kg and 50.78 μg/kg, respectively, and QNLs in pork kidney at 93.36 μg/kg. STr were not detected in any muscle and liver sample from pork and chicken, but it was detected in pork and chicken kidneys. TCs were detected only in pork kidney samples (50%) at a median concentration of 6.89 μg/kg and muscle and liver from chicken at low frequencies (11%).

### 3.4. Exposure Assessment and Risk Characterization

For exposure assessment, we calculated the EDI based on the levels of antibiotics (SAs, TCs, QNLs and STr) in meat samples of three different kinds (pork, bovine and chicken). The daily consumptions per person for Greeks are 41.0 g for bovine, 79.1 g for pork and 70.2 g for poultry meat, according to FAOSTAT. The body weight was considered to be 70 kg [41]. Our results were presented for both methods of analysis used. The EDI through meat consumption (EDIm) of SAs, TCs, QNLs and STr for the Greek population are presented in Table 5 for ELISA values and Table 6 for LC–MS values determined in each kind of meat and total. EDIm did not exceed ADI values either by the type of antibiotic.

Risk characterization methodology, as described by Goumenou and Tsatsakis [31], was applied, in order to assess the risk of exposure to antibiotics (Table 1). Official data were used for the needed calculations. For the risk assessment, we calculated the HQ for each kind of meat and antibiotic (Table 5 and Table 6).

For the ELISA method, the HI was calculated to be 0.059 for QNLs, 0.006 for SAs and 0.005 for STr, lower than the corresponding ADI. We had no detected levels for TCs in the ELISA method. LC–MS results led to the calculation of the HI as 0.011 for ENR and MBX, 0.002 for SMX and 0.0078 for OTC and DOX, still far lower than the corresponding ADIs. Admittedly, there is a big difference between the HI of the two methods applied, proportional to the difference in levels and EDIs.

Risk characterization parameters presented in Table 7 and Table 8 for both methods, reveal that the ratios of cEDIm to ADI are well below the respective CFs for each antibiotic group indicating no risk for the Greek population, with higher values determined for quinolones by ELISA (Table 7). More specifically, normalized results with CF equal to 1, reach a cEDIm/ADI ratio of 0.2863 corresponding to 28.63% risk, expressing the HQ as a percentage of CF.

## 4. Discussion

The results of the present study are compared with similar data in literature in Table 9. A study conducted in southern Italy determined OTC levels in beef muscle and liver samples, using LC–MS [42]. Although the number of samples was greater than the present study, very low frequencies were reported (3% in muscle, 7% in liver). The more positive liver samples compared to muscle and the higher liver concentrations of 31.5 µg/kg (23.9–40.2 µg/kg) compared to muscle concentrations of 15.9 µg/kg (15.0–28.6 µg/kg), show a similar trend that was observed in the present study (83% positive bovine liver samples, range: 66.60–102.1 µg/kg and 30% bovine muscle samples, range: 4.31–17.17 µg/kg).

Higher frequencies as well as higher concentrations may be due to inappropriate use of antibiotics and may depend on the rate of drug administration and amounts used. Oxytetracycline is used for pneumonia and some mouth infections. It has been reported that disease burden can vary between seasons depending on humidity [43]. Furthermore, the rate of metabolism of drugs from the body depends on weather and seasonal variations [44]. It should be noted that the seasons when the samples were collected for the present study were autumn and winter.

Panzenhagen et al. screened ENR in muscles, livers and kidneys from chickens with liquid chromatography [45]. Based on their results, 23% of the muscle samples (mean: 12.3 µg/kg), 17% of liver samples (mean: 45.4 µg/kg) and 17% of kidney samples (mean: 17.4 µg/kg) were positive for ENR. Although higher frequencies of detection (44% in muscle, 33% in liver and 100 in kidney samples) were depicted in the current study, the detected mean values of all type of samples were much lower than those reported in the above study.

**Table 9:** Comparison between current results and data from other monitoring studies in literature.

Reference	Country	Method	N	Samples	Compounds	Mean (μg/kg)	Range	% Positive Samples
**Present study**	Greece	LC–MS	16 beef	Muscle	OTC	10.1	4.3–17.2	30
				Liver		77.5	66.6–102.1	83
**Cammilleri et al., 2019 [42]**	Italy	LC–MS	369 beef	Muscle	OTC	15.9	15.0–28.6	3
				Liver		31.5	23.9–40.2	7
**Present study**	Greece	ELISA	18 pork	Muscle	SAs	6.3	2.8–18.9	100
				Liver		47.2	8.4–86.0	100
				Kidney		14.0	2.5–31.9	83
			20 chicken	Muscle		22.9	1.8–157.3	89
				Liver		4.8	2.5–7.2	100
			16 beef	Muscle		7.4	2.5–30.0	90
				Liver		23.8	2.1–77.5	100
**Ramatla et al., 2017 [46]**	Africa	ELISA	50 pork	Muscle	SAs	0	-	0
				Liver		58.5	48.2–69.9	9
				Kidney		72.7	52.8–92.8	36
			50 chicken	Muscle		47.5	32.5–65.9	12
				Liver		73.4	45.8–81.6	28
			32 beef	Muscle		65.3	-	7
				Liver		51.6	19.8–87.9	29
**Present study**	Greece	LC–MS	20 chicken	Muscle	ENR	3.4	0.4–9.3	44
				Liver		7.8	6.4–9.5	33
				Kidney		2.1	1.4–2.8	100
**Panzenhagen et al., 2016 [45]**	Brazil	LC–MS	72 chicken	Muscle	ENR	12.3	0.96–35.8	23
				Liver		45.4	-	17
				Kidney		17.4	-	17
**Present study**	Greece	ELISA	16 Beef	Muscle	STr	169.8	135.6–191.5	30
**Abdullah et al., 2012 [47]**	Iraq	ELISA	23 Beef	Muscle	STr	59.6	26.0–282.2	61

In South Africa, Ramatla et al. measured sulfonamide residues in pork samples (muscle, liver and kidney) using the ELISA [46]. No sulfonamides were detected in the pork muscle samples, whereas 9% of pork liver samples and 36% of pork kidney samples were positive. The mean concentrations were 58.5 µg/kg (48.2–69.9 µg/kg) and 72.7 µg/kg (52.8–92.8 µg/kg), respectively. The results of the present study are in agreement with Ramatla et al., as higher concentrations of SAs in pork liver/kidney were found compared to pork muscle. In contrast with the literature, higher detection frequencies were found in the present study and particularly all samples of pork muscle were positive.

In a study in Iraq, STr levels in 23 beef muscle samples were determined by ELISA [47]. A total of 61% of the samples were positive with a mean concentration of 59.60 µg/kg (26.0–282.2 µg/kg). However, in our study the results differ significantly as 30% of the samples were positive with a mean concentration of 169.76 µg/kg (135.62–191.5 µg/kg).

The observed differences between the results of the present study and others in literature [46,47] may be due to the way that antibiotics were administered, for example intramuscularly, intravenously or administration via food and drinking water. Furthermore, the long-term use of antibiotics before sampling and the short time between last antibiotic administration and slaughter may be significant parameters for the detection rate of the compounds. According to Yamaguchi et al. [48], the sampling period affected significantly the detected concentrations of antibiotics in chicken samples. Higher or lesser amounts were detected during five separate occasions.

Exposure and risk assessment analysis in the present study showed that the antibiotics levels in chicken, pork and beef from the Cretan market pose no actual risk for human health. To the best of our knowledge, this is the first study for antibiotics in meat from the Greek market although there are others similar in literature. A recent work by Oyedeji et al. [49] presented the concentrations of nineteen antibiotic residues in imported poultry products (turkey muscle and gizzard and chicken muscle) in Nigeria. The risk assessment analysis with the conventional method showed that the dietary exposure to antibiotics per meat type was within safe levels for adults and children. Vragovic et al. examined streptomycin and tetracyclines presence in meat samples of the Croatian market [50]. Similar to the present study, EDI was significantly higher for streptomycin (5.56 μg/person/day or 0.080 μg/kg bw/day) than TCs (0.21 μg/person/day or 0.003 μg/kg bw/day). The same trend was observed in our results too, as performing the LC–MS method for TCs led to EDI approximately two orders of magnitude lower than STr.

In 2017, Wang et al. investigated livestock and poultry meat samples from Shanghai for TCs, QNLs and SAs presence [51]. Estimated daily exposure dose was below 1 μg/kg bw/day, whereas according to the authors aquatic products were a more importance source of these antibiotics than meat or milk. Kyriakides et al. examined the differences in exposure to antibiotics between children and adolescents in Cyprus from the consumption of pork meat for the years from 2012 to 2017 [52]. EDI values were far below ADI and notably higher in children aged 6–9 years old compared to adolescents aged 10–17 years old. All HI values were below 0.056 and indicated low risk exposure for all participants.

A different approach was followed by Zhang et al. [53], who calculated EDI from the urinary levels of the excreted antibiotics to estimate initial exposure of the Chinese. They found that 14.7% of the children had HI greater than 1 as well as 23.6% of the parents and 11.8% of the grandparents, with ciprofloxacin being the major contributor to exposure among all participants. Lately, researchers aimed to describe the antibiotic exposure in Shanghai primary school students [54]. Fluoroquinolones, lincosamides, sulfonamides and tetracyclines were examined and the totally daily exposure dose was found to be below 1 μg/kg bw/day. Finally, the study concluded that intake frequency of white meat (poultry meat) is positively associated with TCS and intake frequency of dairy products with enrofloxacin (QNLs).

## 5. Conclusions

To the best of our knowledge, this is the first study that screened antibiotic residues in bovine, pork and chicken samples (muscle, liver and kidney) from the Greek Cretan market. Only 2% of the samples were free from antibiotics, 2% were detected with 4 antibiotics and the great majority of the samples (87%) were detected with 2 to 3 antibiotics. The risk assessment analysis indicated that there is no risk from beef, pork and chicken consumption corrected for the aggregated exposure. Although intake was estimated to be low and exposure can be considered safe, the dietary habits among consumers vary and increased consumption of several foods that are burdened with antibiotics can raise the risk. Furthermore, low and long-term exposure can have severe effects for gut microbiota which in turn is related with severe consequences for health and diseases that sometimes are not directly correlated with antibiotics exposure.

## 6. Limitations

In the current study, we aimed to determine the levels of four groups of antibiotics in meat samples and to estimate the dietary exposure to antibiotics from meat consumption as well as the potential hazard for human health. Although we tried to address the issue of aggregated exposure through the applied methods, we still have not approached the cumulative exposure issue. Additionally, the local market sampling as well as the consumption data, which were derived from one specific database, limited the scope of objectivity. Finally, each of the two applied methods had its own limitations; the ELISA method provided us with concentration data for a whole group of compounds. In contrast, the LC—MS method offered results for specific compounds, but it was not possible to detect all the compounds of each group.

## Figures and Tables

**Table 1:** Consumption data of food items contributing to the antibiotic dietary exposure, the respective MRLs and the calculated maximum “permitted” daily exposure for each food item (MPDI), for all food items (aggregated (MPDIA)), for meat items estimated (bovine, pig meat and poultry meat) (MPDIm) and correction factor calculated (CF).

		Sulfonamides (SAs)		Tetracyclines (TCs)		Quinolones (QNLs)		Streptomycines (STr)	
Food Item	Consumption Data	MRL	MPDI	MRL	MPDI	MRL	MPDI	MRL	MPDI
	g food/kg bw/day	μg/kg	(μg/kg bw/day)	μg/kg	(μg/kg bw/day)	μg/kg	(μg/kg bw/day)	μg/kg	(μg/kg bw/day)
**Honey**	0.0650	100	0.0065	100	0.0065	100	0.0065		<0.0001
**Bovine Meat**	0.5859	100	0.0586	200	0.1172	100	0.0586	600	0.3515
**Mutton and Goat Meat**	0.3323	100	0.0332	200	0.0665	100	0.0332	600	0.1994
**Pig meat**	1.1299	100	0.1130	200	0.2260	100	0.1130	600	0.6780
**Poultry Meat**	1.0023	100	0.1002	200	0.2005	100	0.1002	600	0.6014
**Meat, Other**	0.0767	100	0.0077	100	0.0077	100	0.0077		<0.0001
**Offals, Edible**	0.1335	100	0.0133	100	0.0133	100	0.0133		<0.0001
**Butter, Ghee**	0.0391	100	0.0039	100	0.0039	100	0.0039		<0.0001
**Cream**	0.0595	100	0.0059	100	0.0059	100	0.0059		<0.0001
**Eggs**	0.3299	100	0.0330	200	0.0660	100	0.0330		<0.0001
**Milk—Excluding Butter**	8.9941	100	0.8994	100	0.8994	100	0.8994	200	1.7988
**Freshwater Fish**	0.0779	100	0.0078	200	0.0156	100	0.0078		<0.0001
**Demersal Fish**	0.1718	100	0.0172	200	0.0344	100	0.0172		<0.0001
**Pelagic Fish**	0.1710	100	0.0171	200	0.0342	100	0.0171		<0.0001
**Marine Fish, Other**	0.0196	100	0.0020	200	0.0039	100	0.0020		<0.0001
**MDPIA (μg/kg bw/day)**			1.3189		1.7009		1.3189		3.6291
**MPDImeat (μg/kg bw/day)**			0.2718		0.5436		0.2718		1.6309
**CFmeat**			0.2061		0.3196		0.2061		0.4494

**Table 2:** Analytical parameters for the applied LC–MS protocol.

	Linearity (r^2^)	LOD (μg/kg)	LOQ (μg/kg)	% Accuracy
**MBX**	0.999	0.06–0.32 *	0.20–1.06 *	101.4
**OTC**	0.967	0.67–1.43 *	2.21–4.72 *	108.9
**ENR**	0.999	0.04–0.14 *	0.13–0.46 *	98.5
**DOX**	0.996	1.02–2.16 *	3.37–7.13 *	94.8
**SDZ**	0.998	2.54	8.38	96.5
**SMX**	0.994	1.15	3.80	92.2

* Depends on the tissue.

**Table 3:** Monitoring results (μg/kg) of antibiotics in all meat samples by LC–MS analysis.

		Bovine		Pork			Chicken		
Compounds	μg/kg	Muscle	Liver	Muscle	Liver	Kidney	Muscle	Liver	Kidney
***SMX***	**% Positive**	40	0	60	50	33	89	22	0
	**Mean ± SD**	22.42 ± 29.78	ND	12.73 ± 5.44	4.49	7.93 ± 4.34	8.23 ± 6.31	4.75 ± 1.12	ND
	**Median**	9.16	ND	12.39	4.49	7.93	5.58	4.75	ND
	**Range**	4.40–66.95	ND	7.23–21.68	ND	4.86–11.00	4.51–22.60	3.96–5.54	ND
***OTC***	**% Positive**	30	83	60	100	100	22	100	0
	**Mean ± SD**	10.1 ± 6.53	77.47 ± 14.37	4.75 ± 2.35	34.80 ± 15.13	10.38 ± 5.13	6.39 ± 1.68	68.57 ± 20.55	ND
	**Median**	8.83	74.46	4.56	34.80	9.08	6.39	64.74	ND
	**Range**	4.31–17.17	66.60–102.06	2.32–8.54	24.10–45.50	5.46–16.69	5.20–7.57	50.16–94.64	ND
***DOX***	**% Positive**	20	0	30	50	50	11	11	0
	**Mean ± SD**	13.28 ± 11.50	ND	53.14 ± 45.51	26.98	99.91 ± 84.81	12.17	31.72	ND
	**Median**	13.28	ND	44.10	26.98	181.73	ND	ND	ND
	**Range**	5.15–21.41	ND	12.84–102.50	ND	12.39–181.73	ND	ND	ND
***ENR***	**% Positive**	20	83	30	50	50	44	33	100
	**Mean ± SD**	3.41 ± 4.24	2.66 ± 1.56	0.56 ± 0.26	1.89	15.63 ± 12.68	3.38 ± 4.20	7.82 ± 1.59	2.10 ± 0.95
	**Median**	3.41	2.66	0.56	ND	21.13	1.88	7.60	2.10
	**Range**	0.41–6.41	0.86–4.69	0.37–0.74	ND	1.12–24.63	0.42–9.34	6.35–9.50	1.43–2.77
***MBX***	**% Positive**	20	0	20	0	100	33	22	0
	**Mean ± SD**	14.71 ± 20.42	ND	0.36	ND	0.86 ± 0.23	1.29	9.12	ND
	**Median**	14.71	ND	ND	ND	0.78	1.29	9.12	ND
	**Range**	0.27–29.15	ND	ND	ND	0.72–1.33	ND	ND	ND

**Table 4:** Monitoring results (in μg/kg) of antibiotics in all meat samples by ELISA analysis.

		Bovine		Pork			Chicken		
Compounds	μg/kg	Muscle	Liver	Muscle	Liver	Kidney	Muscle	Liver	Kidney
**SAs**	**% Positive**	90	100	100	100	83	89	100	100
	**Mean ± SD**	7.38 ± 8.68	23.78 ± 30.11	6.31 ± 4.72	47.22 ± 54.88	14.00 ± 15.19	5.17 ± 4.63	4.82 ± 1.55	4.97 ± 0.89
	**Median**	4.20	9.76	4.40	47.22	3.17	3.60	4.36	4.97
	**Range**	2.52–30.04	2.10–77.51	2.84–18.9	8.41–86.03	2.51–31.89	1.78–15.70	2.54–7.18	4.35–5.60
**STr**	**% Positive**	30	17	0	0	17	0	0	50
	**Mean ± SD**	169.76 ± 29.94	92.47	ND	ND	151.71	ND	ND	53.44
	**Median**	182.10	ND	ND	ND	ND	ND	ND	ND
	**Range**	135.62–191.55	ND	ND	ND	ND	ND	ND	ND
**TCs**	**% Positive**	0	0	0	0	50	11	11	0
	**Mean ± SD**	ND	ND	ND	ND	5.92 ± 1.77	1.97	4.05	ND
	**Median**	ND	ND	ND	ND	6.89	ND	ND	ND
	**Range**	ND	ND	ND	ND	3.88–6.99	ND	ND	ND
**QNLs**	**% Positive**	20	0	0	0	50	44	33	100
	**Mean ± SD**	50.78 ± 40.19	ND	ND	ND	146.82 ± 94.00	52.92 ± 46.54	20.82 ± 10.39	13.44 ± 2.09
	**Median**	50.78	ND	ND	ND	93.36	45.56	20.84	13.44
	**Range**	22.36–79.20	ND	ND	ND	91.74–255.35	12.76–107.80	10.42–31.20	11.96–14.92

**Table 5:** Estimation of the corrected exposure (cEDI) and hazard quotients (HQs) by ELISA detected levels, ADIs and calculated CFs *.

	Quinolones (QNLs)			Sulfonamides (SAs)			Streptomycines (STr)		
	EDI	cEDI	HQ	EDI	cEDI	HQ	EDI	cEDI	HQ
	μg/kg bw/day	μg/kg bw/day		μg/kg bw/day	μg/kg bw/day		μg/kg bw/day	μg/kg bw/day	
**Bovine Meat**	0.0298	0.1444	0.0233	0.0025	0.0119	0.0002	0.1067	0.2374	0.0047
**Pig Meat**	0	0	0	0.0533	0.2589	0.0052	0	0	0
**Poultry Meat**	0.0457	0.2216	0.0357	0.0036	0.0175	0.0004	0	0	0
**cEDIm**		0.3659			0.2883			0.2374	
**HI**			0.0590			0.0058			0.0047
**HI total**	0.078								

* CF_QNLs_ = 0.2061, CF_SAs_ = 0.2061, CF_Str_ = 0.4494, ADI_QNLs_ = 6.2, ADI_SAs_ = 50, ADI_TCs_ = 30, ADI_Str_ = 50 μg/kg bw.

**Table 6:** Estimation of the corrected exposure(cEDI) and hazard quotients (HQs) by LC-MS detected levels (DL), ADIs and calculated CFs *.

Quinolones (QNLs)							Sulfonamides (SAs)			Tetracyclines (TCs)					
	*ENR*			*MBX*			*SMX*			*OTC*			*DOX*		
	EDI	cEDI	HQ	EDI	cEDI	HQ	EDI	cEDI	HQ	EDI	cEDI	HQ	EDI	cEDI	HQ
	μg/kg bw/day	μg/kg bw/day		μg/kg bw/day	μg/kg bw/day		μg/kg bw/day	μg/kg bw/day		μg/kg bw/day	μg/kg bw/day		μg/kg bw/day	μg/kg bw/day	
**Bovine Meat**	0.0020	0.0097	0.0016	0.0086	0.0418	0.0067	0.0054	0.0260	0.0005	0.0051	0.0162	0.0005	0.0078	0.0243	0.0008
**Pig meat**	0.0006	0.0031	0.0005	0	0	0	0.0140	0.0679	0.0014	0.0052	0.0161	0.0005	0.0498	0.1559	0.0052
**Poultry Meat**	0.0019	0.0091	0.0015	0.0013	0.0063	0.0010	0.0056	0.0271	0.0005	0.0064	0.0200	0.0007	0	0	0
**HI**	0.0113						0.0024			0.0078					
**HI total**	0.021														

* CF_QNLs_ = 0.2061, CF_SAs_ = 0.2061, CF_TCs_ = 0.3196, CF_Str_ = 0.4494, ADI_QNLs_ = 6.2, ADI_SAs_ = 50, ADI_TCs_ = 30, ADI_Str_ = 50 μg/kg bw.

**Table 7:** Hazard characterization parameters (by ELISA analysis).

	Sulfonamides (SAs)	Tetracyclines (TCs)	Quinolones (QNLs)	Streptomycines (STr)
**cEDIm (μg/kg bw/day)**	0.288	0.250	0.366	0.237
**ADI (μg/kg bw/day)**	50.000	30.000	6.200	50.000
**HQ (=cEDIm/ADI)**	**0.006**	**0.008**	**0.059**	**0.005**
**MPDIm (μg/kg bw/day)**	0.272	0.544	0.272	1.631
**MPDIA (μg/kg bw/day)**	1.319	1.701	1.319	3.629
**CF**	**0.206**	**0.320**	**0.206**	**0.449**
**Risk %**	**2.798**	**2.605**	**28.637**	**1.111**

**Table 8:** Hazard characterization parameters (by LC–MS analysis).

	Quinolones (QNLs)		Tetracyclines (TCs)		Sulfonamides (SAs)
	ENR	MBX	OTC	DOX	SMX
**cEDIm (μg/kg bw/day)**	0.022	0.048	0.052	0.180	0.121
**ADI (μg/kg bw/day)**	6.200	6.200	30.000	30.000	50.000
**HQs (=cEDIm/ADI)**	**0.004**	**0.008**	**0.002**	**0.006**	**0.002**
**HQ**	0.011		0.008		0.002
**MPDIm (μg/kg bw/day)**	0.272		0.544		0.272
**MPDIA (μg/kg bw/day)**	1.319		1.701		1.319
**CF**	**0.206**		**0.320**		**0.206**
**Risk %**	**5.478**		**2.426**		**0.970**

## Data Availability

Not applicable.
